# Myricitrin Modulates NADPH Oxidase-Dependent ROS Production to Inhibit Endotoxin-Mediated Inflammation by Blocking the JAK/STAT1 and NOX2/p47^phox^ Pathways

**DOI:** 10.1155/2017/9738745

**Published:** 2017-06-20

**Authors:** Shimei Qi, Zunyong Feng, Qiang Li, Zhilin Qi, Yao Zhang

**Affiliations:** ^1^Anhui Province Key Laboratory of Active Biological Macromolecules, Wannan Medical College, Wuhu, China; ^2^Department of Biochemistry, Wannan Medical College, Wuhu 241002, China; ^3^Department of Forensic Medicine, Wannan Medical College, Wuhu, China

## Abstract

Myricitrin, a naturally occurring polyphenol hydroxy flavonoid, has been reported to possess anti-inflammatory properties. However, the precise molecular mechanism of myricitrin's effects on LPS-induced inflammation is unclear. In the present study, myricitrin significantly alleviated acute lung injury in mice. Myricitrin also markedly suppressed the production of NO, TNF-α, IL-6, and MCP-1 in RAW264.7 macrophage cells. The inhibition of NO was concomitant with a decrease in the protein and mRNA levels of iNOS. The phosphorylation of JAKs and STAT-1 was abrogated by myricitrin. Furthermore, myricitrin inhibited the nuclear transfer and DNA binding activity of STAT1. The JAK-specific inhibitor ruxolitinib simulated the anti-inflammatory effect of myricitrin. However, myricitrin had no impact on the MAPK signalling pathway. Myricitrin attenuated the generation of intracellular ROS by inhibiting the assembly of components of the gp91^phox^ and p47^phox^. Suppression of ROS generation using NAC or apocynin or by silencing gp91^phox^ and p47^phox^ all demonstrated that decreasing the level of ROS inhibited the LPS-induced inflammatory response. Collectively, these results confirmed that myricitrin exhibited anti-inflammatory activity by blocking the activation of JAKs and the downstream transcription factor STAT1, which may result from the downregulation of NOX2-dependent ROS production mediated by myricitrin.

## 1. Introduction

Acute lung injury/acute respiratory distress syndrome (ALI/ARDS) refers to a variety of conditions characterized by acute, progressive, hypoxic respiratory failure primarily caused by an inflammatory response rather than cardiogenic factors. Endotoxin or LPS, a component of the cell wall of gram-negative bacteria, is a strong inducer of inflammatory responses. Endotoxaemia caused by a severe gram-negative bacterial infection easily leads to acute lung injury. Clinical studies indicate that the mortality rate from ALI and the more severe form, ARDS, of patients in China varies from 30% to 67.7%, and it is positively correlated with pulmonary inflammation and colloid osmotic pressure in the body [1–3]. Exposure of macrophages to LPS rapidly induces the secretion of proinflammatory mediators, such as NO, prostaglandin E2 (PGE2), ROS, and proinflammatory cytokines, including IL-6, TNF-*α*, and MCP-1. These cytokines further aggravate asthmatic pathological alterations and lung inflammatory responses [4].

Toll-like receptor 4 (TLR4) is the primary pathogen recognition receptor for LPS. LPS bound to TLR4 activates multiple signalling cascades, such as the MAPK as well as Janus kinase and signal transducer and activator of transcription (JAK-STAT) pathways [5–7]. Once LPS binds to TLR4, different JAKs are brought into close proximity and transphosphorylated. Activated JAKs provide docking sites and recruit their primary substrates, the STATs. STATs are consequently phosphorylated and form homo- or heterodimers. Then, they translocate into the nucleus and regulate STAT target genes [8]. In macrophages, STAT1 and STAT3 have been implicated as important transcription factors [9, 10]. For instance, phosphorylation at tyrosine residue 705 on STAT3 is essential for IL-1*β* and IL-6 production in RAW264.7 cells after LPS stimulation [11]. The previous reports have demonstrated that LPS rapidly activates STAT1 through TLR4, and genetic ablation of STAT1 protects against LPS-induced lethality, which suggests that STAT1 may have a key role in LPS-induced inflammation. The STAT1 S727 phosphorylation site selectively regulates TNF-*α* expression in response to stimulation from multiple TLRs [12]. Meanwhile, a lack of induction of IL-6 gene expression is observed in STAT1-deficient mice [13]. After being phosphorylated by JAKs, STATs can regulate the gene expression of acute phase proteins such as MCP-1 and CD40 [14, 15]. In addition, it has been shown that LPS-induced interleukin-1*β* (IL-1*β*) production in macrophages is, in part, regulated through JAK2 [16, 17]. Mitogen-activated protein kinases (MAPKs) are classified into three subfamilies: extracellular signal-regulated kinases 1/2 (ERK1/2), p38, and c-Jun N-terminal kinase (JNK). MAPKs, members of another major inflammatory signalling pathway, play important roles in regulating the expression of several inflammatory genes in various cell types [18]. MAPK-mediated JAK/STAT phosphorylation is important in proinflammatory cytokine-mediated signalling pathways.

ROS refer to a series of highly reactive molecules that include free radicals such as hydroxyl radicals, superoxide, and singlet oxygen, as well as nonradical species, for example, hydrogen peroxide [19–21]. Rather than being simply a by-product of aerobic metabolism, the importance of ROS in innate immunity was first recognized in professional phagocytes undergoing a “respiratory burst” upon activation. It is now recognized that specific enzymes—the NOX and Duox (dual oxidase) enzymes—seem to have the sole function of generating ROS in a carefully regulated manner [22]. In addition, as secondary messengers, ROS participate in cell growth, adhesion, differentiation, senescence, and apoptosis as well as the modification of various signalling molecules [23, 24]. However, excessive production and accumulation of ROS are detrimental to cells and tissues. An unbalanced redox state plays a key role in the development and progression of various inflammatory diseases [25].

The first NOX was found in phagocytic cells involved in the innate immune response. When phagocytes are exposed to bacteria, NOX assembles multiple protein components and generates a rapid increase in the level of ROS [26]. NOX members comprise the catalytic subunits of the membrane-bound proteins gp91^phox^ and p22^phox^ and the four cytosolic proteins p47^phox^, p67^phox^, p40^phox^, and the small GTPase Rac [27]. During full activation of the NADPH oxidase, at least three cytosolic subunits, p47^phox^, p67^phox^, and p40^phox^, form a complex and translocate to the membrane, where they integrate with the gp91^phox^-p22^phox^ complex. The gp91^phox^ catalytic subunit and the p47^phox^ regulatory subunit play a key role in acute activation of NADPH oxidase [28]. Phosphorylation of p47^phox^ breaks away intracellular inhibitory components and tends to bind to gp91^phox^-p22^phox^ complex, thereby increasing NADPH oxidase activation [29]. Our previous studies demonstrated that ROS are upstream signalling molecules induced during the inflammatory response in RAW264.7 cells through AKT and NF-*κ*B pathways [30]. Apocynin, an NOX inhibitor, has protective effects against LPS-induced ALI in rats [31]. In THP-1 cells and primary human monocytes, an IRAK-dependent p67^phox^-NOX2 interaction induced by LPS-mediated TLR4 activation promoted ROS generation. Eliminating intracellular ROS inhibits IL-1*β* transcription and processing [32]. Moreover, STAT1 signalling plays a critical role in intracellular redox signalling in activated macrophages [33]. NOX2-dependent ROS regulate the macrophage immune response via gp91^phox^ and p47^phox^, interacting with TLR3 and activating STAT1 phosphorylation and then promoting inflammatory mediator release [34].

**Figure 1:**
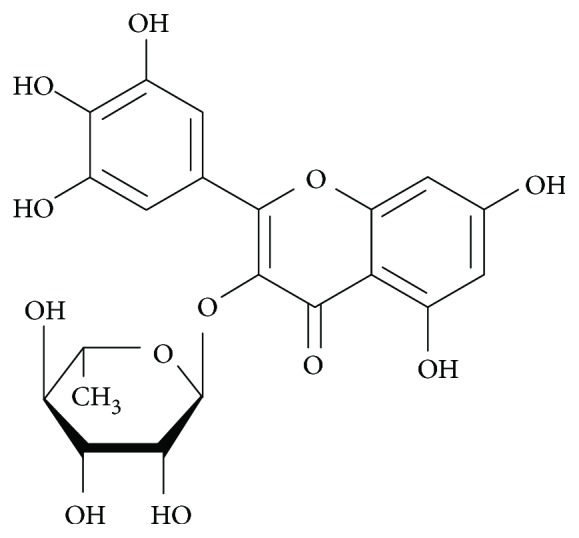
Chemical structure of myricitrin (C21H20O12, molecular weight = 464.3763).

Myricitrin (3′, 4′, 5′, 5, 7-five hydroxyflavone-3-O-*α*-L-rhamnoside) (Figure 1) is a naturally occurring polyphenol hydroxy flavonoid that is abundant in bayberry fruits, branches, bark, and leaves, as well as in other varieties of plants [35, 36]. Myricitrin has a variety of beneficial properties, such as antiviral, antimicrobial [37], antinociceptive [38], and anticarcinogenic [39, 40] activities. In particular, myricitrin possesses stronger oxidative resistance and free radical scavenging activity than other flavonol rhamnosides or quercetin [41]. In addition, the potent antioxidative activities of myricitrin may be attributed to its polyhydroxy structure. The previous reports have shown that myricitrin can suppress acrylamide-induced cytotoxicity by inhibiting ROS production [42]. Myeloperoxidase generates excess oxidants, which cause oxidative stress and oxidative tissue damage. Myricitrin can irreversibly inactivate myeloperoxidase activity [43]. Shimosaki et al. confirmed that myricitrin inhibited TNF-*α* production in RAW264.7 macrophages [44]. Moreover, myricitrin reduced iNOS expression and the production of its product, NO, induced by LPS [45].

In spite of studies indicating that myricitrin has antioxidant activity and anti-inflammatory potential, only a few studies have focused on whether myricitrin can influence signalling pathways involved in the inflammatory response in LPS-activated macrophages. The specific molecular mechanism involved in myricitrin scavenging of intracellular LPS-induced ROS has not been fully elucidated. In the present study, a mouse model of LPS-induced ALI was employed to assess the potential anti-inflammatory effects of myricitrin. To explore the molecular mechanisms, we also evaluated its effect on MAPK and JAK/STAT activation. The role of gp91^phox^/p47^phox^ activation in ROS production and the anti-inflammatory effect of myricitrin were further investigated.

## 2. Materials and Methods

### 2.1. Antibodies and Reagents

Myricitrin was obtained from Aladdin Industrial Corporation (Shanghai, China). Monoclonal and polyclonal antibodies against iNOS, COX-2, JNK, phospho-JNK (Thr183/Tyr185), p38, phospho-p38 (Thr180/Tyr182), ERK1/2, phospho-ERK1/2 (Thr202/Tyr204), JAK1, phospho-JAK1 (Tyr1022/1023), JAK2, phospho-JAK2 (Tyr1007/1008), STAT1, phospho-STAT1 (Tyr701), STAT3, phospho-STAT3 (Tyr705), phospho-STAT3 (Ser727), TBP, gp91^phox^, Na/K ATPase-*α*1, and GAPDH were purchased from Cell Signaling Technology (Beverly, MA, USA). Antibody to p47^phox^ was obtained from Santa Cruz Biotechnology (CA, USA). All secondary antibodies used for western blotting were purchased from LI-COR Biosciences (Lincoln, NE, USA). LPS (from *Escherichia coli* 0111:B4), NAC, and DAPI were obtained from Sigma-Aldrich (St. Louis, MO, USA). CCK-8 was purchased from KeyGen Biotech (Nanjing, JS, China). CM-H2DCFDA was obtained from Invitrogen (Carlsbad, CA, USA). Ruxolitinib and apocynin were purchased from Selleck Chemicals (Houston, TX, USA). All ELISA kits were purchased from R&D Systems China Co. Ltd. (Shanghai, China).

### 2.2. DNA Constructs and RNA Interference

Small hairpin RNA (shRNA) constructs against p47^phox^ mediated by a pFU-GW-007 shRNA vector (Ncf-1, GenBank_ID: NM_001286037), shRNA constructs against gp91^phox^ mediated by a pFU-GW-007 shRNA vector (Cybb, GenBank_ID: NM_007807), and pFU-GW-007 as a negative control were constructed by Ji Kai Gene Chemical Technology (Shanghai, China). All of the constructs were verified by DNA sequencing. RAW264.7 cells were transfected with shRNA or negative control using polyethylenimine (PEI) transfection reagent (Sigma-Aldrich, St. Louis, MO, USA) according to the manufacturer's instructions. Interference efficiency was confirmed by immunoblot analysis after 72 h of transfection using p47^phox^ and gp91^phox^ antibody.

### 2.3. Cell Culture and Transfection

RAW264.7 cells derived from murine macrophages (ATCC number: TIB-71) were obtained from the Institute of Kunming Cell Bank, the Chinese Academy of Sciences (Kunming, YN, China). RAW264.7 cells were cultured in Dulbecco's modified Eagle's medium (DMEM, HyClone, Logan, UT, USA) containing 10% fetal bovine serum (HyClone, Logan, UT, USA) at 37°C in a 5% CO_2_ incubator. Transient transfection was performed with PEI transfection reagent (Sigma-Aldrich, St. Louis, MO, USA) according to the manufacturer's instructions. In all cases, the total amount of DNA was normalized by empty control plasmids.

### 2.4. Cell Viability Assay

RAW264.7 cells seeded at 1 × 10^4^ cells/well in 96-well plates were treated with myricitrin of different concentrations (0, 10, 50, 100, 150, 200, 250, 300, 400, and 500 *μ*g/ml). For the blank group, only cell culture medium was added. After 24 h, the culture medium was replaced and 10 *μ*l of CCK-8 reagent was added. Then, cells were incubated at 37°C for 2 h and slightly mixed. The absorbance (*A*) at 450 nm was measured using a Thermo Multiskan GO Universal Microplate Reader (Waltham, USA). Cell viability was calculated using the following formula: Cell viability (%) = (*A* (dosing)–*A* (blank))/(*A* ( dosing)–*A* (blank))∗100.

### 2.5. Reactive Oxygen Species (ROS) Detection

The intracellular accumulation of ROS, including H_2_O_2_ and other peroxides, was monitored using the fluorescent probe CM-H2DCFA. At the end of the treatment, cells were loaded with 10 *μ*M CM-H2DCFA and incubated at 37°C for 30 min in the dark. Cells were then rinsed and resuspended in PBS. Samples were examined using an Olympus IX51 fluorescence microscope (Tokyo, Japan).

### 2.6. Cytokine Measurement

RAW264.7 cells seeded at 2 × 10^5^ cells/well in 12-well plates were treated as indicated in the figure legends. Cultured medium was collected and centrifuged at 10,000 rpm for 5 min. Cytokine levels (PGE2, IL-6, TNF-*α*, and MCP-1) in the culture supernatant were determined using commercially available ELISA kits (R&D Systems), according to the manufacturer's protocol.

### 2.7. Nitrite Analysis

The accumulation of nitrite, the stable metabolite of NO, in the culture medium was measured as an indicator of NO production. Levels of nitrite in the culture media were measured using a commercially available Griess assay kit (KeyGen Biotech, Nanjing, JS, China) according to the manufacturer's instructions. Absorbance at 550 nm was measured, and nitrite concentrations were calculated by comparison with standard solutions of sodium nitrite.

### 2.8. Isolation of Subcellular Fractions

Cell stimulation was terminated by the addition of ice-cold PBS. The nuclear, cytosolic, and membrane protein extracts were prepared using a nuclear and cytoplasmic protein extraction kit and a membrane and cytosol protein extraction kit (Beyotime, Shanghai, China) according to the manufacturer's instructions. All steps of the subcellular fractionation were performed at 4°C. Fraction purity was tested by western blotting using GAPDH as the cytoplasmic marker, TBP as the nuclear marker, and Na/K ATPase-*α*1 as the membrane marker.

### 2.9. Western Blotting

Cells were rinsed twice with ice-cold PBS and solubilized in RIPA lysis buffer (Beyotime, Shanghai, China) containing a protease inhibitor (Roche, Basel, Switzerland) for 30 min on ice. Lysates were centrifuged (15,000 ×g) at 4°C for 10 min. Equal amounts of the soluble protein were denatured in SDS, electrophoresed on an 8–12% SDS-PAGE gel, and transferred to nitrocellulose membranes (Millipore, Boston, MA, USA). Transferred proteins were incubated with the corresponding primary antibodies at 4°C overnight. After extensive washing (three times for 5 min each in Tris-buffered saline with Tween 20 (TBST)), proteins were detected by incubation with IRDye 800-conjugated IgG secondary antibodies (LI-COR Biosciences, Lincoln, NE, USA) at room temperature for 1 h. The proteins were visualized using a LI-COR Odyssey infrared imaging system (Lincoln, USA).

### 2.10. Quantitative Real-Time Reverse Transcription Polymerase Chain Reaction (qRT-PCR) and RT-PCR

Total RNA was extracted from treated cells using TRIzol reagent (Invitrogen, Paisley, Scotland) and used to synthesize cDNA using a Thermo Scientific RevertAid First Strand cDNA Synthesis Kit (Thermo, USA) according to the manufacturer's instructions. The qPCR was performed using Thermo Maxima SYBR-Green/ROX qPCR Master Mix (Waltham, USA). The primer sequences were as follows: 5′-GGGTCTTGTTCACTCCACGG-3′ (forward) and 5′-GCTCAGAACAGCACAAGGGG-3′ (reverse) for iNOS and 5′-CTGACCCCCAAGGCTCAAAT-3′ (forward) and 5′-GGGGATACACCTCTCCACCA-3′ (reverse) for COX-2. The relative amount of iNOS and COX-2 mRNA species was compared to that of GAPDH and calculated using the 2^−△△Ct^ data analysis method. Each Ct value used for the calculations was the mean of three experiments performed for each reaction. mRNA expression was normalized to the housekeeping gene GAPDH. The primer pairs used for PCR were the same as those used for qRT-PCR. PCR products were resolved on 1.5% agarose gels and were stained with GoldView. RT-PCR was used to verify the effectiveness and specificity of primers.

### 2.11. Electrophoretic Mobility Shift Assays (EMSAs)

Nuclear proteins were extracted using a nuclear and cytoplasmic protein extraction kit (Beyotime, Shanghai, China) according to the manufacturer's instructions. Protein was quantified by the BCA method (Beyotime, Shanghai, China). All extracts were stored at −80°C until use. A DNA-binding assay was performed on nuclear extracts using a biotin probe-labelling EMSA kit (Beyotime, Shanghai, China) according the manufacturer's instructions. The STAT1 double-stranded oligonucleotide probe sequence used in this study was 5′ biotin-CATGTTATGCATATTCCTGTAAGTG-biotin3′, which was synthesized by GenScript (Nanjing, China). The binding reaction was performed in a 10 *μ*l mixture containing 4 *μ*l of nuclease-free water, 2 *μ*l of EMSA/gel-shift binding buffer, 2 *μ*g of nuclear extracts, and 1 *μ*l of the indicated probes. To ensure the specificity of the binding, we simultaneously prepared unlabelled and mutant probe groups along with the supershift group. After a 30 min incubation at room temperature, 1 *μ*l of EMSA/gel-shift loading buffer was added and the samples were electrophoresed through a 6.5% nondenaturing polyacrylamide gel at 150 V in an ice bath for 2 h. Then, the samples were transferred to a nylon membrane and cross-linked for 15 min on a UV transilluminator at 312 nm. Biotin-labelled DNA-protein complexes were detected by a Vilber Quantum-ST5 gel imaging system (Beijing, China) and photographed.

### 2.12. Immunofluorescence Staining and Confocal Microscopy

To study the subcellular distribution of signalling molecules, RAW264.7 cells were sequentially immunostained, first with primary antibodies against p-STAT1, p47^phox^, or gp91^phox^ and then with the appropriate Alexa Fluor 555-conjugated or Alexa Fluor 488-conjugated secondary antibody. Briefly, RAW264.7 cells were fixed in 4% paraformaldehyde in PBS for 15 min at room temperature, permeabilized with 0.1% Triton X-100 in PBS for 5 min, and then blocked for 1 h in PBS containing 3% bovine serum albumin. Cells were incubated with primary antibody for 1 h at room temperature in blocking buffer. After rinsing 3 times in PBS solution, samples were incubated with the appropriate secondary antibodies. Slides were counterstained with 0.1 *μ*g/ml DAPI. Images were captured with a Leica TCS SP8 confocal laser microscope system (Heidelberg, Germany).

### 2.13. Animals

Male BALB/c mice (6–8 weeks old, 20–25 g) obtained from Vital River Inc. (Beijing, China) were maintained in pathogen-free environments in the animal centre of our college. The mice were kept in a temperature-controlled room with a standard 12 h light/dark cycle. Food and drinking water were available ad libitum. Before the experiments started, the mice underwent an acclimatization period of at least 7 days. All the procedures in the animal experiments were approved by the Chinese Experimental Animals Administration Legislation and performed strictly according to the Guide for the Animal Care and Use Committee of Wannan Medical College. All operations were performed under anaesthesia with chloral hydrate (3.5% chloral hydrate, 0.35 g/kg, i.p.). All efforts were made to minimize suffering of the animals.

### 2.14. Establishment of an Acute Lung Injury (ALI) Model

An ALI model was set up in mice by intratracheal injection of LPS. Briefly, mice were anaesthetised with 0.35 g/kg of chloral hydrate, and then, they received an intratracheal instillation of 100 *μ*g of LPS in 50 *μ*l of sterile saline using a 3-gauge needle. Then, the mice were placed in a vertical position and rotated for 1 min to distribute the instillation in the lungs. Forty male BALB/c mice were randomly divided into 4 groups (*n* = 10): control group, dexamethasone (0.5 mg/kg) group, ALI group, and myricitrin (120 mg/kg) treatment group. The mice in the control group only received 50 *μ*l of sterile saline. In the dexamethasone group (0.5 mg/kg) and myricitrin (120 mg/kg) treatment group, mice were treated with dexamethasone and myricitrin for 4 h via penis vein injection prior to the LPS challenge. Twelve hours after the LPS challenge, mice were sacrificed for further analysis.

### 2.15. Histopathological Analysis

Twelve hours after LPS administration, the mice were anaesthetised with 0.35 g/kg of chloral hydrate and decapitated and bled from the neck to prevent alveolar congestion. After priming the lung tissue with 4% paraformaldehyde and tying up the tracheal opening, the lung tissue of each mouse was fixed in 4% paraformaldehyde, embedded in paraffin, and cut into 5 *μ*m thick sections. Following hematoxylin and eosin (H&E) staining according to the regular staining method, the pathological alterations in the lung tissues were evaluated under a light microscope by an experienced observer, and then, photomicrographs were taken.

### 2.16. Statistical Analysis

Values are presented as the mean ± SD. Statistical analysis was performed by Student's *t*-test and one-way ANOVA. SPSS 13.0 software was used to calculate *P* values. Each value of *P* < 0.05 was considered statistically significant.

## 3. Results

### 3.1. Effect of Myricitrin on the Production of Proinflammatory Mediators and Cytokines

**Figure 2:**
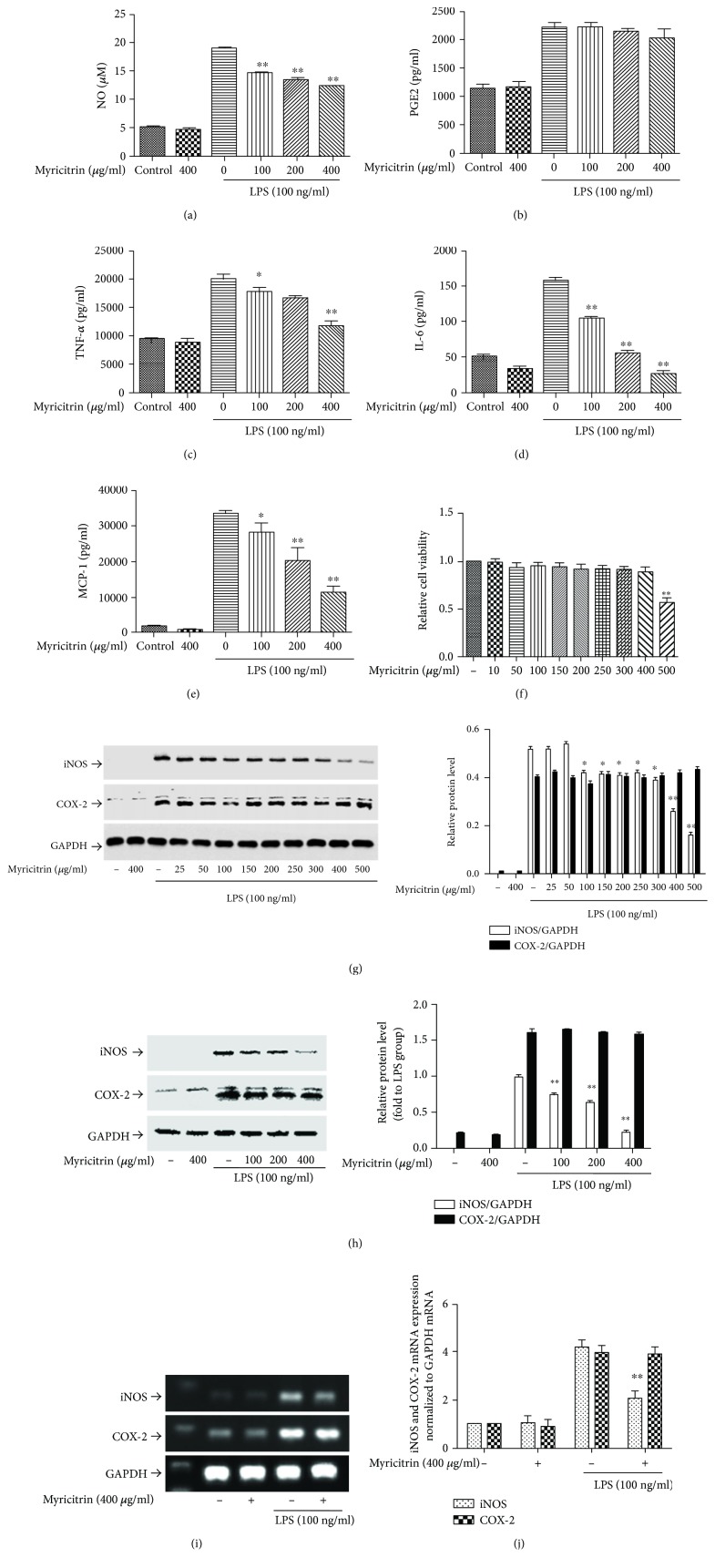
Noncytotoxic level of myricitrin inhibited LPS-induced inflammatory-associated cytokine and mediator production. RAW264.7 cells were treated with myricitrin (100, 200, and 400 *μ*g/ml) or vehicle for 2 h. Next, cells were stimulated with LPS (100 ng/ml) for 16 h. (a) The supernatants were taken, and the amounts of NO were measured by Griess reagents. (b–e) The levels of PGE2, TNF-*α*, IL-6, and MCP-1 were measured in the culture medium by ELISA kits. (f) RAW264.7 cells were treated with indicated concentration of myricitrin for 24 h. Cell viability was evaluated using the CCK-8 assay, and the results were expressed as percentage of surviving cells over the control group. RAW264.7 cells were incubated with 100, 200, and 400 *μ*g/ml of myricitrin or vehicle for 2 h and then were stimulated with LPS (100 ng/ml). (g–h) After incubation of 16 h, cell lysates were prepared and subjected to western blotting by using anti-iNOS and anti-COX-2 antibodies. GAPDH was the internal control. After incubation of 8 h, total RNA was isolated and iNOS and COX-2 mRNA were determined by RT-PCR (i) and qRT-PCR (j). Each bar represents the mean ± SD of three independent experiments. ^∗^*P* < 0.05 and ^∗∗^*P* < 0.01 versus LPS-stimulated groups.

NO and PGE2 are important inflammatory mediators generated at sites of inflammation. Our previous studies showed that in LPS-stimulated RAW264.7 cells, the levels of NO and PGE2 were substantially increased [30]. To evaluate the anti-inflammatory effects of myricitrin, we first measured the production of NO and PGE2 in RAW264.7 cells following LPS treatment. Our results showed that LPS triggered an obvious increase in NO and PGE2 levels in the culture media. Myricitrin suppressed NO production in a concentration-dependent manner, but had no effect on PGE2 (Figures 2(a) and 2(b)). It has been reported that LPS induces NO production by increasing the expression of iNOS, and COX-2 is the inducible enzyme of PGE2 [46–48]. Therefore, we detected the protein and mRNA levels of iNOS and COX-2. As we expected, myricitrin decreased LPS-induced iNOS expression. COX-2 levels exhibited no significant changes after treatment, which was consistent with the PGE2 results (Figures 2(g)–2(j)). LPS can induce RAW264.7 cells to generate large amounts of proinflammatory cytokines [49, 50]. Thus, we measured the release of IL-6, TNF-*α*, and MCP-1. Different doses of myricitrin alone had no effect on cytokine production; however, myricitrin markedly suppressed IL-6, TNF-*α*, and MCP-1 production in the LPS-stimulated group (Figures 2(c)–2(e)). To exclude the possibility that cytotoxic activity of myricitrin caused inhibition of inflammation-associated mediators and cytokines, we tested cell viability with a CCK-8 assay. The results indicated that within the range of different doses used, cell viability was above 95%, which showed that myricitrin was not cytotoxic to RAW264.7 cells (Figure 2(f)). These results suggested that myricitrin inhibited the LPS-related inflammatory response in RAW264.7 cells.

### 3.2. Effect of Myricitrin on LPS-Induced JAK/STAT1 and MAPK Phosphorylation

**Figure 3:**
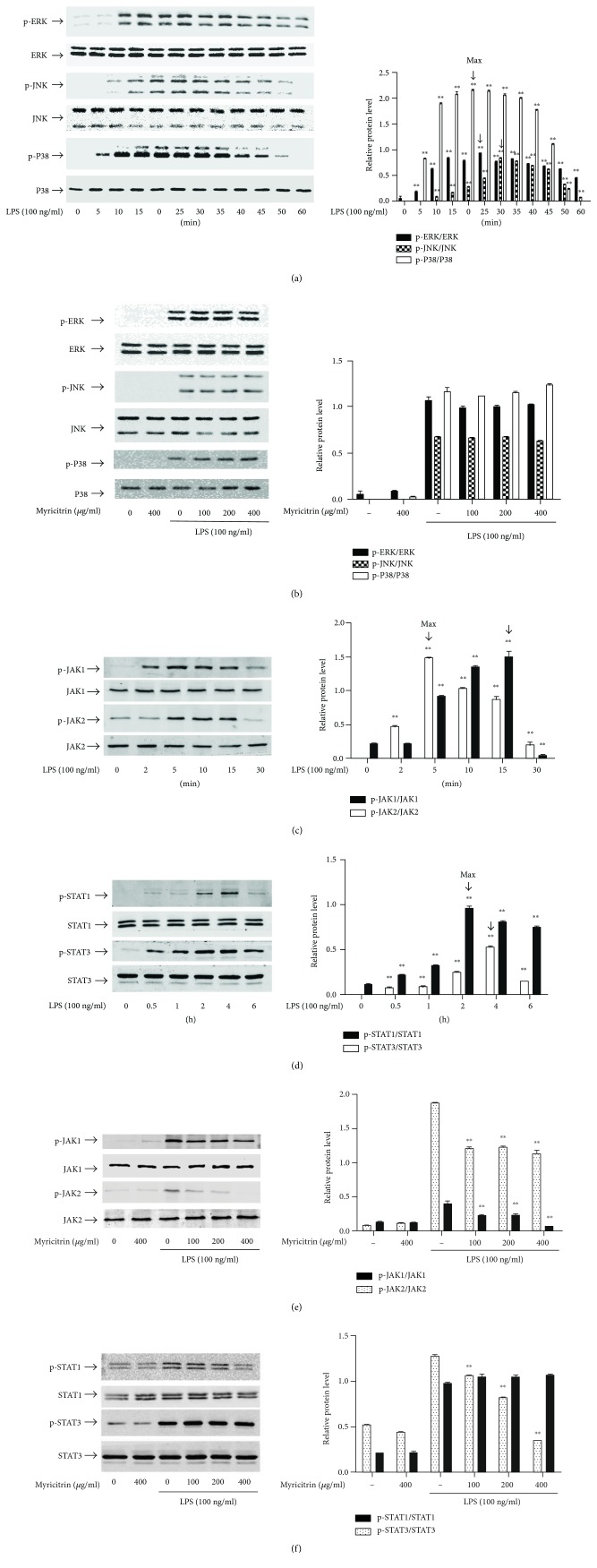
Noncytotoxic level of myricitrin inhibited LPS-induced JAK and STAT activation but had no impact on MAPKs phosphorylation. RAW264.7 cells were stimulated with LPS for (a) 0, 5, 10, 15, 20, 25, 30, 35, 40, 45, 50, and 60 min; (c) 0, 2, 5, 10, 15, and 30 min; and (d) 0, 0.5, 1, 2, 4, and 6 h. In addition, RAW264.7 cells were pretreated with myricitrin at 100, 200, and 400 *μ*g/ml for 2 h and then stimulated with LPS for 30 min (b), 15 min (e), and 4 h (f). Total protein was subjected to 10% SDS-PAGE followed by western blotting using specific antibodies against phospho-ERK, phospho-JNK, and phospho-P38 (a, b); phospho-JAK1 and phospho-JAK2 (c, e); and phospho-STAT1 and phospho-STAT3 (d, f). Nonphosphorylated antibodies were the internal control. Each value indicated the mean ± SD and was a representative of the results obtained from three individual experiments. ^∗∗^*P* < 0.01 versus LPS-stimulated groups.

**Figure 4:**
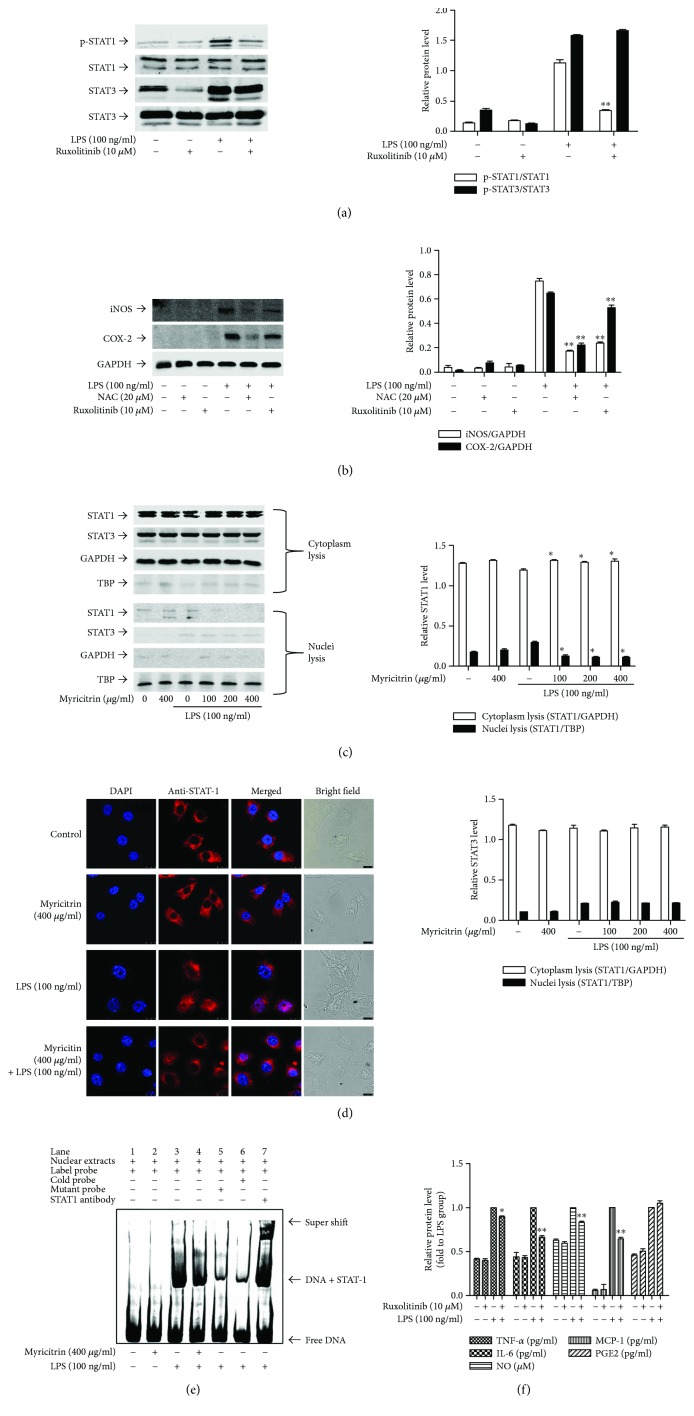
Noncytotoxic level of myricitrin inhibited LPS-induced JAK activation, which was required for LPS-induced STAT phosphorylation and nuclear translocation as well as inflammation-related substances. (a) RAW264.7 cells were treated with LPS (100 ng/ml) for 6 h in the presence or absence of 10 *μ*M ruxolitinib for 2 h. Phosphorylation of STAT1 and STAT3 was measured by western blotting analysis using specific antibodies. (b) RAW264.7 cells were pretreated with 10 *μ*M ruxolitinib or 20 *μ*M NAC for 2 h and stimulated with 100 ng/ml LPS for 16 h. Expression levels of iNOS and COX-2 protein were determined by immunoblotting. RAW264.7 cells were pretreated with the indicated concentrations of myricitrin for 2 h before incubation with LPS (100 ng/ml) for 6 h. (c) The nuclear and cytoplasm proteins were extracted. Equal amounts of protein were subjected to immunoblot analysis with antibodies against STAT1 and STAT3. GAPDH was used as the cytoplasmic internal control and TBP was used as the nucleus internal control. (d) Immunostaining was performed with STAT1 (in red) and nuclei were stained with DAPI (in blue). Scale bars: 10 *μ*m. Nuclear translocation of STAT1 was observed under a fluorescence microscope. (e) Nuclear extracts were analyzed for STAT1 activity by EMSA in the presence or absence of excess amounts of cold probe, mutant probe, or STAT1 antibody. (f) RAW264.7 cells were treated with ruxolitinib (10 *μ*M) for 2 h. Next, cells were stimulated with LPS (100 ng/ml) for 16 h. Levels of TNF-*α*, IL-6, MCP-1, and PGE2 in culture supernatants were determined by ELISA. The amounts of NO were measured by Griess reagents. The data obtained from three different areas were mean ± SD. One of the representative data obtained from three individual experiments was shown. ^∗^*P* < 0.05 and ^∗∗^*P* < 0.01 were compared with the LPS group.

Recent studies have provided evidence that LPS activates MAPK (ERK1/2, p38, and JNK1/2) and JAK/STAT signalling pathways and thereby causes intracellular inflammation [51, 52]. To clarify the mechanisms underlying the anti-inflammatory effects of myricitrin, we examined whether myricitrin affected MAPK and JAK/STAT signalling activation.  Figure 3(a) shows that phosphorylation of MAPKs induced by LPS peaked at 30 min and was sustained until 60 min. However, pretreatment with myricitrin had no obvious impact on the LPS-triggered activation of MAPK signalling molecules (Figure 3(b)). Then, we evaluated the role of STAT signalling activation in the myricitrin-mediated anti-inflammatory effect. As demonstrated in (Figure 3(d)), STAT1 and STAT3 activation was initiated at 0.5 h, peaked at 4 h, and weakened at 6 h. Thus, in subsequent experiments, we used the 4 h time point as the stimulation time of STATs. Myricitrin treatment significantly suppressed LPS-stimulated STAT1 phosphorylation. However, there were no visible changes in p-STAT3 after myricitrin treatment (Figure 3(f)). Phosphorylated STAT proteins form a dimer, translocate to the nucleus, bind to specific DNA elements, and thus regulate the transcription of thousands of genes [53]. We extracted cytoplasmic and nuclear proteins. The results of western blotting showed that the protein expression of nuclear STAT1 increased after treatment with LPS and this response was inhibited in all myricitrin groups. As expected, LPS promoted STAT3 translocation into the nucleus, but myricitrin could not reverse the change (Figure 4(c)). Confocal microscopy was performed to further observe the nuclear localization of STAT1. As shown in Figure 4(d), in control and myricitrin alone groups, STAT1 (red) was scattered in the cytoplasm. In the LPS treatment group, STAT1 entered the nucleus and merged (pink) with DAPI (blue). Myricitrin pretreatment inhibited STAT1 nuclear translocation. To measure the transcriptional regulatory activity of STAT1, we conducted EMSA with the corresponding biotin-labelled consensus sequences of STAT1. We found increased binding activity of STAT1 in nuclear extracts prepared from the LPS treatment group. The enhanced binding affinity was significantly inhibited by myricitrin (Figure 4(e)).

JAK1 and JAK2 are the upstream kinases of STATs. LPS induced the activation of JAK1 and JAK2 from 2 min to 30 min, and the activation peaked at 5 min (Figure 3(c)). This response of JAK2 was dramatically blocked by the treatment with myricitrin in a dose-dependent manner. p-JAK1 levels were less changed than those of p-JAK2 after myricitrin treatment (Figure 3(e)). To further determine the anti-inflammatory effects mediated by myricitrin via inhibition of the JAKs/STAT1 signalling pathway, we explored whether the JAK-specific inhibitor ruxolitinib could simulate the anti-inflammatory effect of myricitrin. We found that 10 *μ*M ruxolitinib could effectively inhibit phosphorylation of the downstream signalling molecules STAT1 and STAT3 (Figure 4(a)). The results showed that ruxolitinib could markedly inhibit iNOS expression and weakly restrain COX-2 expression (Figure 4(b)). In addition, the production of the inflammatory cytokines IL-6, TNF-*α*, and MCP-1, as well as the inflammatory mediator NO, was clearly suppressed by ruxolitinib (Figure 4(f)). In general, myricitrin exerted anti-inflammatory effects by inhibiting JAK/STAT1 activation, preventing phosphorylation of STAT1, which prevented it from entering the nucleus and reduced the transcriptional activity of STAT1.

### 3.3. Effect of Myricitrin on LPS-Induced ROS Production

**Figure 5:**
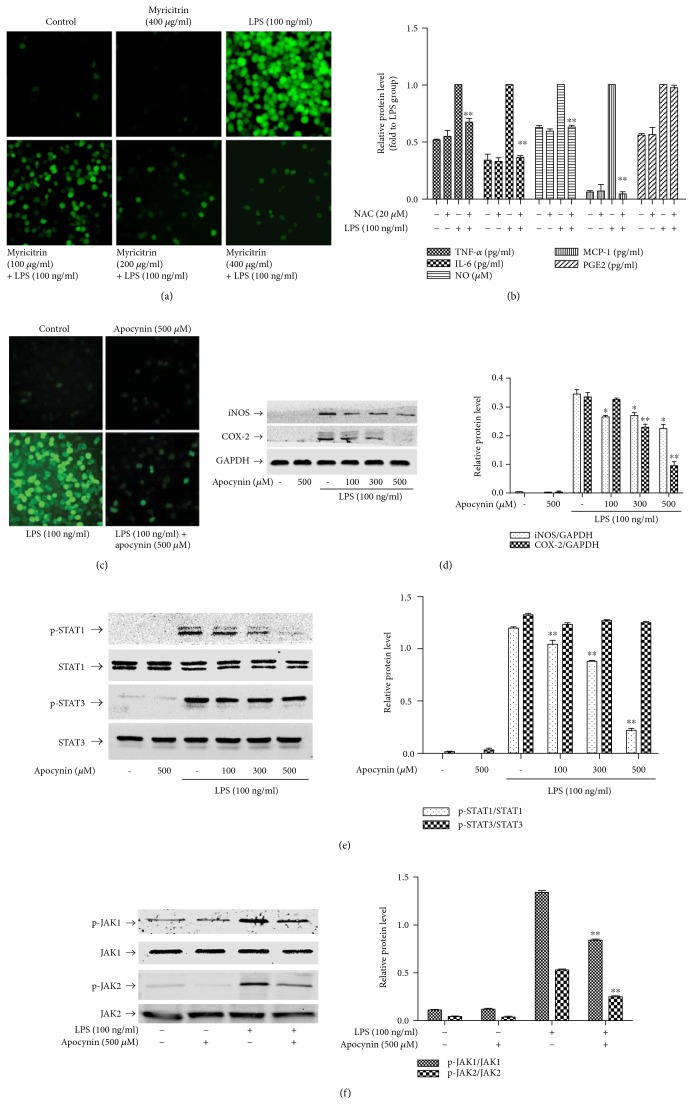
Noncytotoxic level of myricitrin inhibited NOX2-derived ROS generation, whose ROS was required for LPS-induced JAK/STAT1 activation as well as inflammatory cytokine and mediator production. (a) RAW264.7 cells were pretreated with myricitrin for 2 h and then exposed to LPS (100 ng/ml) for 5 min. After treatment, cells were washed with PBS and the fluorescence intensity was measured by flow cytometry as described in Materials and Methods. (b) RAW264.7 cells were incubated with or without NAC (20 *μ*M) for 2 h and then stimulated with LPS (100 ng/ml) for 16 h. Culture media were assayed for NO production with the Griess reaction and for TNF-*α*, IL-6, MCP-1, and PGE2 production by ELISA. (c) RAW264.7 cells were pretreated with apocynin (500 *μ*M) for 2 h and then exposed to LPS (100 ng/ml) for 5 min. The treatment of cells was the same as (a). RAW264.7 cells were incubated with apocynin (500 *μ*M) for 2 h and then stimulated with 100 ng/ml LPS for 16 h (d), 4 h (e), and 15 min (f). Cells were harvested, and equal amounts of whole cell lysates were analyzed by western blotting with anti-iNOS or anti-COX-2 antibody (d), p-STAT1 or p-STAT3 antibody (e), and p-JAK1 or p-JAK2 antibody (f). Western blot detection of GAPDH or nonphosphorylated antibodies was estimated as the protein-loading control for each lane. Data were mean ± SD values of three independent experiments. ^∗^*P* < 0.05 and ^∗∗^*P* < 0.01 versus LPS-treated group.

Excessive ROS levels can induce the inflammatory response in RAW264.7 cells through activation of transcription factors, including NF-*κ*B and STATs [30, 54]. To ascertain the role of ROS in LPS-triggered inflammation and the relationship between ROS and myricitrin, we measured ROS production using the fluorescent probe CM-H2DCFA. As seen in Figure 5(a), LPS stimulation generated large amounts of ROS, which were attenuated after myricitrin treatment. Furthermore, pretreatment of RAW264.7 cells with N-acetyl-L-cysteine (NAC), an ROS scavenger, significantly inhibited LPS-induced iNOS and COX-2 protein expression (Figure 4(b)). As shown in Figure 5(b), NAC also decreased IL-6, TNF-*α*, MCP-1, and NO production, while showing a weak impact on the production of PGE2. Thus, myricitrin likely inhibits LPS-associated inflammatory responses by preventing intracellular ROS generation in LPS-stimulated RAW264.7 cells.

### 3.4. Effect of Myricitrin on LPS-Stimulated NADPH Oxidase Activity

**Figure 6:**
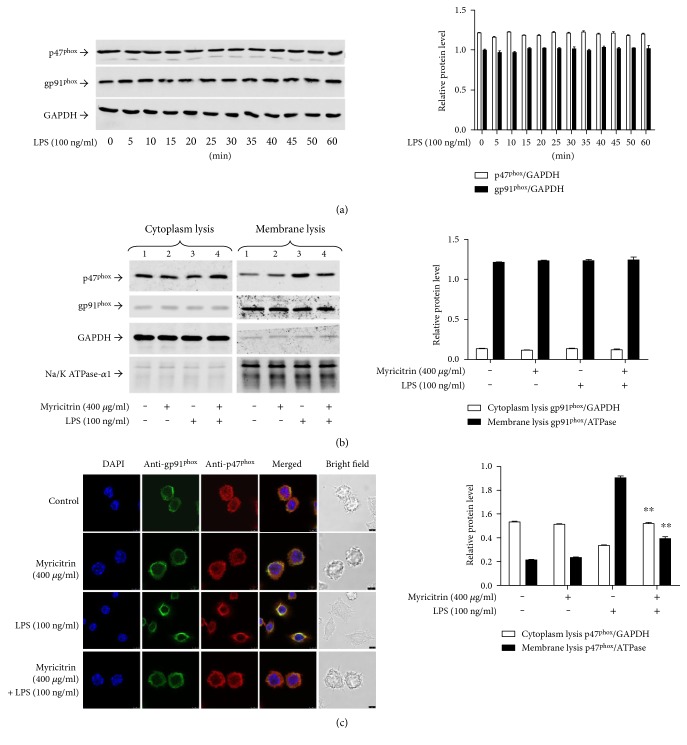
Noncytotoxic level of myricitrin inhibited p47^phox^ which was transferred to the membrane and suppressed the interaction between p47^phox^ and gp91^phox^. (a) RAW264.7 cells were treated with LPS (100 ng/ml) for indicated time points. Cell lysates were prepared and subjected to western blotting by using p47^phox^ and gp91^phox^. (b) RAW264.7 cells were pretreated with 400 *μ*g/ml myricitrin for 2 h or not and then were incubated with LPS (100 ng/ml) for 15 min. The cytosolic and membrane fractions were analyzed for detection of p47^phox^ and gp91^phox^ by western blotting analysis. (c) RAW264.7 cells were treated as similar as (b). Double immunostainings were performed with anti-gp91^phox^ (in green) and anti-p47^phox^ (in red); nuclei were stained with DAPI (blue). Scale bars: 10 *μ*m. The results were expressed as mean ± SD of three independent experiments. ^∗∗^*P* < 0.01 versus LPS-treated group.

NADPH oxidases are the primary source of cellular ROS, generated in response to xenobiotics, cytokines, and bacterial invasion [55, 56].  Figure 5(c) shows that the NADPH oxidase-specific inhibitor apocynin significantly blocked the LPS-induced ROS generation. Moreover, pretreatment with apocynin significantly reduced iNOS and COX-2 expression (Figure 5(d)) and inhibited phosphorylation of JAKs and STAT1 (Figures 5(e) and 5(f)). The results prompted us to assess whether NADPH oxidases were the target by which myricitrin inhibited the inflammatory response. First, we explored the changes in NADPH oxidases before and after LPS treatment. The p47^phox^ regulatory subunit and the membrane catalytic subunit gp91^phox^ play critical roles in activation of NADPH oxidases [57]. However, during the period of LPS treatment, the protein levels of p47^phox^ and gp91^phox^ exhibited no obvious changes (Figure 6(a)). The previous studies have confirmed that upon cell activation, the cytosolic component p47^phox^ is phosphorylated and migrates to the plasma membrane, where it associates with gp91^phox^ to activate NADPH oxidases, which produce superoxide anions [57, 58]. Thus, we observed the subcellular localization of p47^phox^ and gp91^phox^. Our results demonstrated that LPS induced a decrease in the p47^phox^ levels in cytoplasmic lysates, and myricitrin reversed this trend. Simultaneously, in the membrane lysates, LPS treatment led to an increase in p47^phox^, and myricitrin reduced the p47^phox^ content in these fractions. gp91^phox^ was present in the membrane lysates from each experimental group (Figure 6(b)). These results implied that LPS treatment could prompt p47^phox^ transfer from the cytoplasm to the membrane. Next, we further detected the position of NADPH oxidase subunits using confocal laser microscopy. As shown in Figure 6(c), in the resting state, p47^phox^ was scattered in the cytoplasm and gp91^phox^ was distributed in the membrane. There was no overlap between them. Upon LPS stimulation, p47^phox^ was relocated to the membrane and colocalized with gp91^phox^, which is highlighted in yellow. Myricitrin pretreatment completely reversed this change. The results indicated that activated p47^phox^ translocated to the plasma membrane and associated with the gp91^phox^ subunit to assemble into an active enzyme complex.

**Figure 7:**
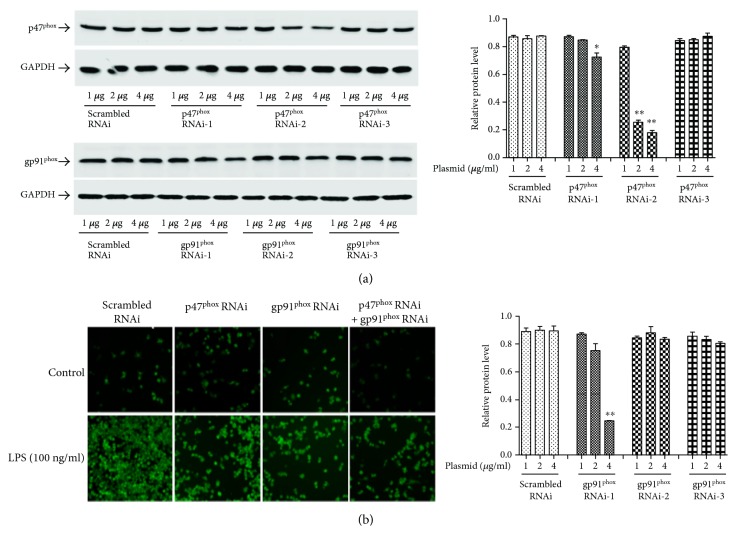
p47^phox^ and gp91^phox^ RNAi inhibited the generation of intracellular ROS. (a) RAW264.7 cells were transiently transfected with 1 *μ*g, 2 *μ*g, or 4 *μ*g pFU-GW-p47^phox^, pFU-GW-gp91^phox^ shRNA plasmid, or pFU-GW plasmid vector, respectively. After 48 h transfection, cell lysates were subjected to immunoblotting using antibodies against p47^phox^ and gp91^phox^. GAPDH was used as the loading control. (b) RAW264.7 cells were, respectively, transfected with shRNA-p47^phox^ and shRNA-gp91^phox^ or cotransfected with shRNA-p47^phox^ and shRNA-gp91^phox^. In the scrambled RNAi group, cells were transfected with pFU-GW plasmid vector. After 48 h transfection, cells were stimulated with LPS (100 ng/ml) for 5 min. After treatment, cells were washed with PBS and the fluorescence intensity was measured by flow cytometry as described in Materials and Methods. These experiments were independently repeated for three times and the results were expressed as mean ± SD. ^∗^*P* < 0.05 and ^∗∗^*P* < 0.01 versus scrambled RNAi group.

**Figure 8:**
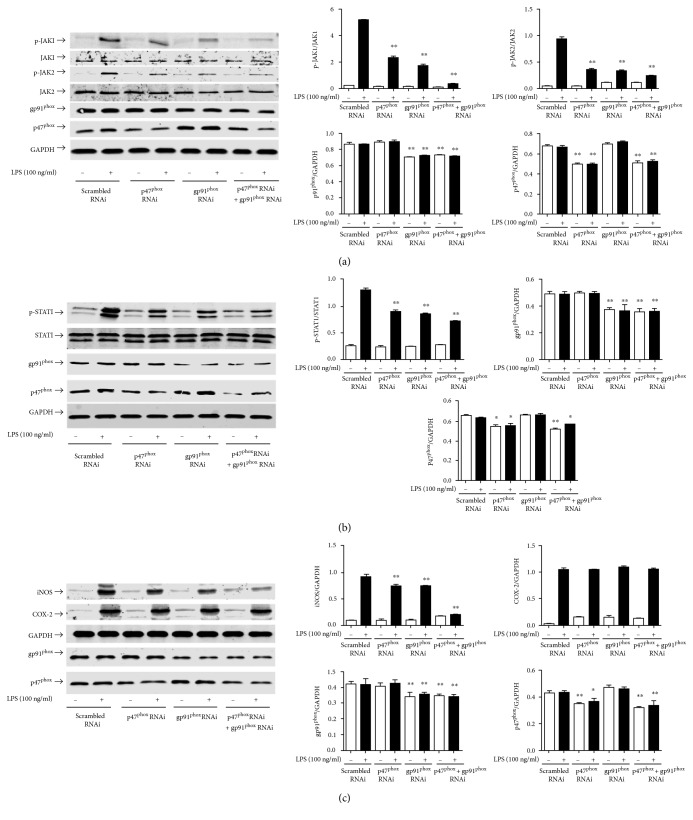
p47^phox^ and gp91^phox^ RNAi inhibited LPS-induced JAK/STAT1 activation and decreased iNOS and COX-2 expression. RAW264.7 cells were, respectively, transfected with shRNA targeting p47^phox^ or gp91^phox^ and cotransfected with p47^phox^ and gp91^phox^ shRNA. The scrambled RNAi group was transfected with pFU-GW plasmid vector. Forty-eight hours after transfection, RAW264.7 cells were stimulated with LPS (100 ng/ml) for 15 min (a), 4 h (b), and 16 h (c). Cell lysates were prepared and subjected to western blotting by using phospho-JAK1 and phospho-JAK2 antibodies (a), phospho-STAT1 antibody (b), and anti-iNOS and anti-COX-2 antibodies (c). GAPDH and nonphosphorylated antibodies were estimated as the protein-loading control for each lane. p47^phox^ and gp91^phox^ were estimated to identify the effect of interference. Each bar represents the mean ± SD of three independent experiments. ^∗^*P* < 0.05 and ^∗∗^*P* < 0.01 versus scrambled RNAi group.

To further clarify the role of NAPDH oxidases in LPS-initiated inflammation and to determine whether ROS are the upstream target molecules through which myricitrin regulates intracellular inflammation, we constructed three shRNA groups with interference plasmids of p47^phox^ and gp91^phox^ and detected the interference efficiency.  Figure 7(a) shows that p47^phox^ shRNA2 effectively suppressed p47^phox^ expression. In the 4 *μ*g p47^phox^ shRNA2 transfected group, the inhibition rate achieved 80%. Meanwhile, gp91^phox^ shRNA1 knocked down endogenous gp91^phox^ expression. Transfection with 4 *μ*g gp91^phox^ shRNA1 plasmids into RAW264.7 cells inhibited approximately 70% of the gp91^phox^ expression. In subsequent experiments, we selected the above interference conditions. As expected, in the LPS test groups, the level of ROS production clearly increased following LPS treatment. p47^phox^ or gp91^phox^ shRNA single transfection significantly reduced the intracellular ROS level compared to the scrambled shRNA transfection. p47^phox^ and gp91^phox^ shRNA cotransfection had an even better inhibitory effect. In the control groups, transfection of RAW264.7 cells individually with scrambled shRNA, p47^phox^ shRNA or gp91^phox^ shRNA, had no impact on the basal ROS expression level (Figure 7(b)). An immunoblot analysis showed that p47^phox^ or gp91^phox^ knockdown reduced LPS-triggered iNOS expression and JAK/STAT1 phosphorylation, while simultaneous knockdown of p47^phox^ and gp91^phox^ achieved the best inhibitory effect (Figures 8(a), 8(b), and 8(c)).

### 3.5. Effect of Myricitrin on LPS-Induced Acute Lung Injury (ALI) in Mice

**Figure 9:**
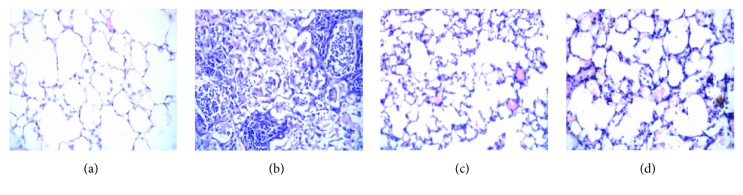
Effects of myricitrin on histopathological changes in the lung tissues of the mice with LPS-induced acute lung injury (ALI). Mice received a single dose of intratracheal instillation of 50 *μ*l saline with or without (5 mg/kg) LPS, and some of LPS-exposed mice were pretreated with myricitrin (120 mg/kg) or dexamethasone (0.5 mg/kg) via penis vein injection for 4 h. Twelve hours after LPS challenge, mice were euthanized with diethyl ether. The whole lung tissue of mice was primed with paraformaldehyde. The lungs from each group were processed for histological evaluation at 12 h after LPS challenge. (a) Control group; (b) LPS (5 mg/kg) group; (c) dexamethasone (0.5 mg/kg) + LPS (5 mg/kg); (d) myricitrin (0.5 mg/kg) + LPS (5 mg/kg). Representative histological changes of lungs obtained from mice of different groups are shown here (hematoxylin and eosin staining, magnification: 400x).

LPS inhalation is a widely used model of acute lung injury (ALI), which is characterized by infiltration of inflammatory cells, interalveolar septal thickening, pulmonary oedema, patchy haemorrhage, hyaline membrane formation, exudation in the alveolar cavity, and some collapsed alveoli [59–61]. To evaluate the effects of myricitrin on ALI, histological changes in the lung tissues were investigated by H&E staining 12 h after LPS inhalation. As illustrated in Figure 9, the lung tissues in the control animals exhibited consistent alveolar lobule structural integrity and a clean alveolar cavity, without haemorrhage or effusion in the alveolar spaces. In the lungs of model animals, the LPS injection caused significant inflammatory cell infiltration in the airway and alveolar space, which indicated the success of the ALI model. However, these pathological alterations induced by LPS were markedly attenuated by myricitrin treatment. Dexamethasone is a steroid anti-inflammatory medication that is used to treat bacterial pneumonia and has widespread clinical use. In our experiment, we used dexamethasone as a positive control drug to assess the anti-inflammatory effect of myricitrin. Figure 9 shows that dexamethasone greatly ameliorated the pathological changes in ALI. Myricitrin has a therapeutic effect similar to dexamethasone. Therefore, myricitrin has a great potential as a clinical anti-inflammatory medication.

## 4. Discussion and Conclusion

The activation of macrophages through excessive production of various proinflammatory cytokines and mediators, reactive oxygen species, and reactive nitrogen species, all of which further exacerbate inflammation, is known to play a central role in inflammation and host defence mechanisms [62]. In this study, we confirmed that myricitrin inhibited the production of NO, TNF-*α*, IL-6, and MCP-1 in LPS-stimulated RAW264.7 macrophage cells. In vivo, we also demonstrated that myricitrin relieved acute lung injury in mice and inhibited LPS-induced inflammatory responses in macrophages. Myricitrin markedly reduced the infiltration of inflammatory cells in lung tissues. In addition, myricitrin decreased the mRNA and protein level of iNOS, which is the catalytic enzyme that produces NO. This demonstrated that the effect of myricitrin on NO production was due to its ability to reduce iNOS mRNA generation and then inhibit iNOS protein expression. These results indicate that myricitrin has anti-inflammatory effects, but the precise mechanism of action has not been fully elucidated.

STAT1 undergoes rapid phosphorylation in response to stimulation by multiple TLR ligands. Phosphorylated STAT1 directly recruits TLR4 and regulates genes involved in the production of proinflammatory cytokines and mediators, which suggests a key role for STAT1 in TLR4-induced inflammation [12]. Our results showed that myricitrin inhibited LPS-induced STAT1 phosphorylation in a dose-dependent manner. We further demonstrated that myricitrin suppressed phosphorylation of JAK1/2. Myricitrin also inhibited nuclear translocation and DNA binding of STAT1 in LPS-activated RAW264.7 cells. Ruxolitinib, a specific JAK1/2 inhibitor, completely simulated the anti-inflammatory effect of myricitrin, including inhibition of TNF-*α*, MCP-1, IL-6, and NO production, as well as STAT1 activation and iNOS protein generation. These results suggest that myricitrin exerts anti-inflammatory effects via STAT1 signalling through mechanisms involving upstream targets, resulting in nuclear translocation and DNA binding of STAT1.

STAT1, STAT3, and STAT4 have a conserved MAPK phosphorylation site, Pro-X-Ser-Pro. Treatment of cells with IFN-*β* causes MAPKs to physically interact with the alpha subunit of STAT1. All of these data imply that there may be a functional communication between MAPK and JAK/STAT signalling pathways [63–65]. In our studies, LPS induced phosphorylation of MAPKs, but myricitrin pretreatment had no effect on the activation of MAPKs. The cytokines IL-2 and IL-6 appear to regulate STAT phosphorylation in a MAPK-independent fashion [8]. Thus, we speculated that in LPS-stimulated RAW264.7 cells, the activation of STAT1 did not pass through the MAPK pathway.

Emerging evidence suggests that LPS stimulates TLR4 and promotes the production of ROS in macrophages [66]. Despite their well-established oxidation activities, recent studies have shown that ROS act as a second messenger upstream of diverse biological responses involving TLR-induced innate immune responses, including autophagy and inflammation [24, 67, 68]. We observed that the level of intracellular ROS increased sharply following LPS treatment. Different concentrations of myricitrin effectively reduced the amount of ROS generated. In the highest dose group, ROS levels dropped to background level. After treatment with NAC, an ROS scavenger, clearing the intracellular ROS, the LPS-induced release of TNF-*α*, IL-6, MCP-1, and NO, as well as the iNOS expression, were inhibited. We speculate that myricitrin may exert anti-inflammatory effects by regulating the intracellular level of oxidative stress.

NOX2-dependent ROS generation is required for TLR4-dependent inflammatory responses in macrophages [69]. The subunits gp91^phox^ and p47^phox^ are essential components of NADPH oxidase. Our findings indicated that myricitrin had no effect on the protein expression level of gp91^phox^ and p47^phox^. ROS generation did not seem to be related to a reduced NOX expression but was more likely related to reduced activity. Further studies confirmed that p47^phox^ was recruited into the cell membrane and bound with gp91^phox^ after LPS treatment. Meanwhile, myricitrin completely reversed the action of LPS. Apocynin, a NOX2-specific inhibitor, scavenges intracellular ROS and decreases the production of proinflammatory cytokines and mediators. Apocynin also inhibited the activation of JAK1/2 and STAT1. In contrast to myricitrin, apocynin significantly inhibited COX-2 expression. To exclude nonspecific effects of apocynin, we knocked down p47^phox^ and gp91^phox^. In cells separately transfected with p47^phox^ or gp91^phox^, LPS-induced intracellular ROS accumulation, JAKs/STAT1 activation, and iNOS expression were weakened. The p47^phox^ and gp91^phox^ cotransfection group achieved the best inhibitory effect. The protein level of COX-2 showed no significant change after transfection. In addition, myricitrin had no obvious effect on the LPS-triggered activation of STAT3. It has been reported that STAT3 is primarily distributed in mitochondria and regulated by mitochondrial peroxide under oxidative stress [70]. We speculate that myricitrin mainly adjusted NOX-derived ROS levels, while STAT3 was influenced by mitochondria peroxide. The discrepancy in the change in COX-2 between myricitrin, apocynin, and RNAi treatments, as well as the different effects of myricitrin on STAT1 and STAT3, further confirmed that the target of myricitrin was the NOX enzyme complex.

In brief, as shown in Figure 0(), LPS-boosted ROS generation was essential for the activation of JAK1/JAK2, promoting STAT1 phosphorylation and nuclear translocation, which plays a key role in the release of inflammatory mediators and cytokines. Myricitrin blocked JAKs/STAT1 signalling transduction by scavenging intracellular ROS. Furthermore, myricitrin inhibited the assembly of the components of the NOX enzyme complex (gp91^phox^ and p47^phox^). The critical role of NOX-derived ROS in myricitrin regulation of LPS-induced inflammation suggested that myricitrin has the potential to become an anti-inflammatory drug possessing a specific targeting ability.

## Figures and Tables

**Figure 10:**
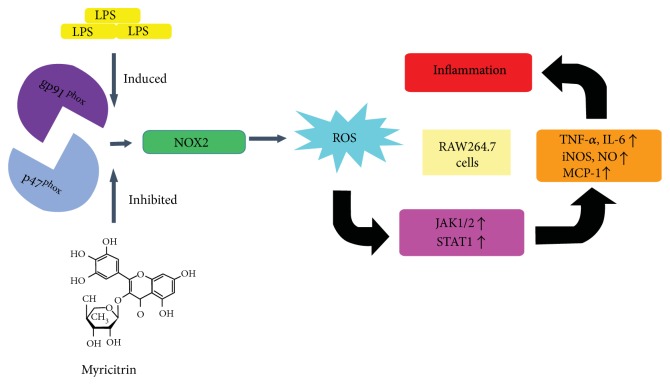
Schematic diagram illustrating the proposed signalling pathway involved in myricitrin's inhibition of LPS-induced inflammation-associated proteins in RAW264.7 cells. LPS activated the NOX2 (gp91^phox^/p47^phox^) pathway to enhance ROS generation, which in turn initiated the activation of JAK1, JAK2, and STAT1 and ultimately induced TNF-*α*, IL-6, MCP-1, and NO production and iNOS expression in RAW264.7 cells. Moreover, pretreatment with myricitrin inhibited LPS-induced inflammation via gp91^phox^/p47^phox^ inhibition.
